# Detection of Hydrogen Peroxide in Liquid and Vapors Using Titanium(IV)-Based Test Strips and Low-Cost Hardware

**DOI:** 10.3390/s22176635

**Published:** 2022-09-02

**Authors:** Rayhan Hossain, Jimmy J. Dickinson, Allen Apblett, Nicholas F. Materer

**Affiliations:** 1 Department of Chemistry, Oklahoma State University, 107 Physical Sciences, Stillwater, OK 74078, USA; 2 Engineering, Physical Sciences, and Process Technology Division, Northern Oklahoma College, Stillwater, OK 74078, USA

**Keywords:** titania, peroxide vapor, thin film sensor, colorimetry

## Abstract

Titanium(IV) solutions are known to detect hydrogen peroxide in solutions by a colorimetric method. Xplosafe’s XploSens PS commercial titanium(IV)-based peroxide detection test strips are used to detect hydrogen peroxide in liquids. The use of these test strips as gas-phase detectors for peroxides was tested using low-cost hardware. The exposure of these strips to hydrogen peroxide liquid or gas leads to the development of an intense yellow color. For liquids, a digital single-lens reflex camera was used to quantify the color change using standardized solutions containing between 50 and 500 ppm peroxide by mass. Analysis of the images with color separation can provide a more quantitative determination than visual comparison to a color chart. For hydrogen peroxide gas, an inexpensive web camera and a tungsten lamp were used to measure the reflected light intensity as a function of exposure from a test strip held in a custom cell. First-order behavior in the color change with time was observed during the exposure to peroxide vapor over a range of peroxide concentrations from 2 and 30 ppm by volume. For a 1-min measurement, the gas-phase detection limit is estimated to be 1 ppm. A 0.01 ppm detection limit can be obtained with a 1-h exposure time. Titanium(IV)-based peroxide detection test strips are sensitive enough to work as a gas-phase hydrogen peroxide detector.

## 1. Introduction

Peroxides (O_2_^2−^) are extremely strong oxidizing agents of great importance in many industrial, chemical, and biological fields. Of the common peroxides, hydrogen peroxide (H_2_O_2_) is used as a bleaching agent in the paper and textile industries [1,2] and as a reagent for the treatment of organic and other pollutants in water supplies [3,4]. In sterile environments, where no bacterial or microbial growth can be tolerated, gaseous hydrogen peroxide is often used as a cleaning reagent [5,6,7,8,9,10,11]. Due to its strong oxidizing potential, the Occupational Safety and Health Administration (OSHA) has set the 8-h day permissible average exposure limit at 1 ppm [12]. Thus, industrial users must be able to accurately monitor background levels of hydrogen peroxide.

Hydrogen peroxide detection and quantification methods include fluorometric, electrochemical, and chemiresistive measurements [13,14,15,16,17,18,19,20,21,22,23,24,25]. Many of these methods cannot be easily automated because they require pre-concentration of the atmosphere in solution, some amount of wet chemistry, or require constant attention by a technician. The OSHA VI-6 method uses the color change of an acidified titanium(IV) solution to colorimetrically quantify hydrogen peroxide vapors by bubbling a known volume through this solution [26]. Other methods include the use of Dräger or similar tubes specific for hydrogen peroxide [27]. Except for Dräger tubes and their like, most commercial hydrogen peroxide gas-phase detectors are amperometric electrochemical cells. Commercial colorimetric methods for liquids often use an enzyme-based method that employs horseradish peroxidase. The colorimetric enzyme-free titanium(IV) solution has the advantage of not requiring a liquid medium.

**Figure 1:**
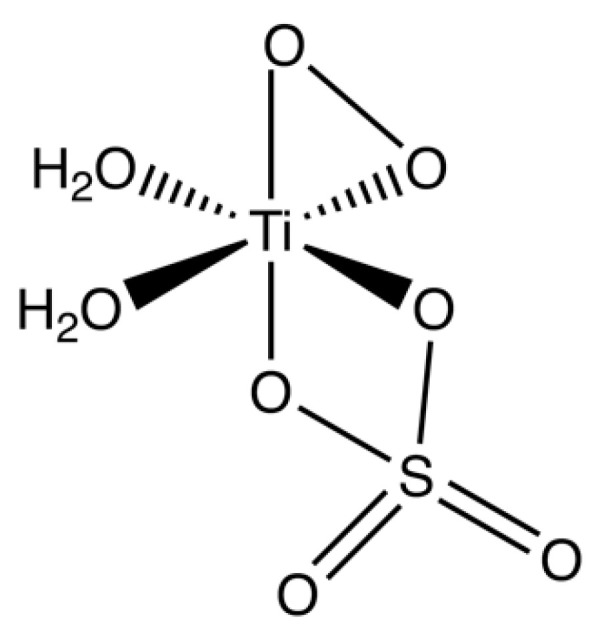
The suggested mononuclear complex with a peroxide dianion chelating to a titanium center (see text for more information).

The titanium(IV) compound used for detection of peroxides is composed of Ti-O-Ti-O-zig-zag chains coordinated to water and hydrogen sulfate ions [28]. When reacted with hydrogen peroxide below pH 1, a mononuclear complex with one peroxide anion per titanium ion forms [29]. The mononuclear complex has been characterization by infrared spectroscopy, and the data suggests a structure similar to the one shown in Figure 1, where a peroxide dianion chelates to the titanium center [30]. Although the crystal structure of the titanium peroxide complex has yet to be determined, there are structurally characterized titanium(IV) complexes with similar chelated peroxide groups that are also deeply orange in color [31]. Given a mononuclear complex, chemical reaction describing the detection stoichiometry can be proposed, as shown in Equation (1).

H_2_O_2_ + TiOSO_4_xH_2_O → [Ti(O_2_)SO_4_]xH_2_O + H_2_O (1)

The color that develops is due to complexation of peroxide by titanium(IV) leading to a ligand to metal charge transfer band at 410 nm that gives the complex its characteristic intense color. As this specific charge transfer band can only form with peroxide complexation, the color formation is extremely selective. Furthermore, color formation is not due to a redox process. Thus, the reagent does not respond to other strong oxidants such as ozone or nitrogen dioxide. Taken together, the selectivity, stability and final intense color of the resulting titanium(IV)-peroxide complexes are the main reasons behind these materials being used for colorimetric detection of peroxides in solutions. The lack of reversibility allows a robust measurement of the final color.

The current work complements previous studies by investigating the use of titanium(IV) oxysulfate confined on a commercially-available test strip. For example, the cellulose paper method based on aqueous titanium(IV)-oxo complexes has been presented by Zang and co-workers [32]. Materer and coworkers have also used a sol-gel approach to create a nanoparticle titanium dioxide (i.e., titania nanoparticles) within a hydroxypropyl cellulose thin film for hydrogen peroxide gas sensing [33]. This paper extends these studies to show that titanium(IV) oxysulfate can be used as non-aqueous gas-phase detection method with high sensitivity using a low-cost optical detector. Although the robust nature of the titanium(IV) complex requires a new detection strip after each measurement, it nevertheless allows the material to perform a passive time integrated exposure measurement without additional hardware.

## 2. Materials and Methods

### 2.1. Peroxide Solutions

**Figure 2:**
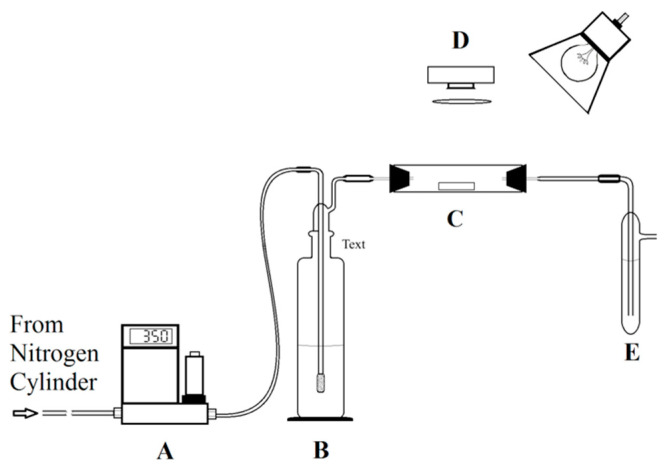
A schematic diagram of the apparatus depicting the (**A**) flow controller, (**B**) bubbler to entrain peroxide vapor, (**C**) exposure chamber, (**D**) detection system, and (**E**) a bubbler used to determine the total concentration of peroxide in the flow.

Peroxide solutions of various concentrations were made through dilution of a commercially available 30% hydrogen peroxide solution (BDH). Hydrogen peroxide solutions with concentrations ranging from 500 to 10 ppm by mass were prepared by first preparing a 1000 ppm stock solution assuming a concentration of 30% for the reagent solution. The actual peroxide concentration of this initial solution was determined by three separate iodometric titrations using a sodium thiosulfate solution. A stock 0.10 N aqueous sodium thiosulfate was purchased (VWR Chemicals BDH, BDH72281) and diluted by a factor of 10 or 100 as necessary. Using the determined concentration, the stock hydrogen peroxide solution was diluted to 500, 350, 250 and 100 ppm. These solutions were analyzed and confirmed to be within 1% of the nominal value. The 100 ppm solution was next diluted to 50 ppm and the resulting solution was again titrated to confirm the correct concentration. Peroxide solutions were also produced for entraining hydrogen peroxide in the gas phase. Solutions with numerous concentrations (1, 2, 5, 10, 15, and 20% hydrogen peroxide by volume) were made by dilution of 30% stock solution and stored in a refrigerator at 4 °C when not being used. When used, each solution was warmed to room temperature over the course of one hour and then poured into Bubbler B as shown in Figure 2. The solution phase concentrations used for Bubbler B were not measured by titration. However, the resulting gas phase concentrations were measured for each experiment (See Figure 2 and discussion below).

### 2.2. Titanium(IV) Oxysulfate Solution

Measurement of the vapor concentration of hydrogen peroxide in the gas phase was performed according to an older OSHA method that utilizes a bubbler to capture hydrogen peroxide rather than quartz filters [13]. The older method was chosen because it has half the standard error (2.9%) than the newer method (5.8%) and is more convenient in the laboratory. This procedure uses a colorimetric reagent prepared by adding 5.5 g of titanium(IV) oxysulfate (TiOSO_4_xH_2_O, purchased from Strem Chemicals and dried overnight in a vacuum desiccator) and 20 g of ammonium sulfate (Spectrum, A.C.S. Reagent) to 100 mL of concentrated sulfuric acid (Pharmco-Aaper, A.C.S. Reagent). The mixture was then heated carefully on a hotplate with stirring until the solid reagents had dissolved. The cooled solution was then diluted with 350 mL of ultrapure water and the resulting solution was filtered through a 0.40 µm glass-fiber filter to remove any undissolved solids that might lead to turbidity. The filtered solution was then diluted to 500 mL.

### 2.3. Testing Apparatus

Figure 2 shows a schematic diagram of the apparatus that is used to expose and monitor the color of the test strips. Except as noted, the apparatus is similar to the system reported previously [33]. Briefly, the nitrogen cylinder (Airgas, Ultrahigh Purity) was used to supply nitrogen as the carrier gas at 350 standard cubic centimeters per minute (sccm) using an Omega FMA 5500 mass flow controller. Next, the nitrogen flow was passed through a 250 mL bubbler containing peroxide solution (labeled B) and dispersed using a sintered glass head. The solution concentration of hydrogen peroxide in the bubbler determines the gas phase concentration, which is directly measured as described below.

**Figure 3:**
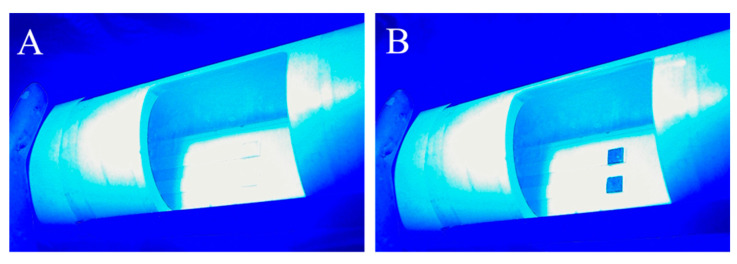
A reflected image from two XploSafe PS test-strips (**A**) before and (**B**) after exposure to a hydrogen peroxide gas at a concentration of 28 ppm for one hour.

After the bubbler, the peroxide-enriched gas was directed into a custom PTFE (Polytetrafluoroethylene) hollow cylinder (labeled C) containing the test strip. Control experiments were performed where the hydrogen peroxide concentration was measured before and after the glass and PTFE tubes to ensure that there was no loss in the apparatus. Previous work utilizing a glass tube showed some reactivity with the cylinder wall leading to a few percent error in the gas-phase hydrogen peroxide concentration. For organic peroxides, the glass tube was unacceptably reactive. The PTFE used in the current experiments was found to be significantly less reactive towards hydrogen peroxide and organic peroxides, making it the material of choice that eliminated a potential source of error. The PTFE cylinder had a small cutout (see Figure 3) that was used to allow light to enter and exit the cell and was sealed by thin PTFE film. For the gas concentration measurement (see below), the cell must be leak-tight to ensure that all gas entering the cell exits and passes through the exit bubbler (labeled E). Within the cell, the test strips rested on a small PTFE shelf in the center of the tube.

A light source (a 20 W incandescent bulb) was positioned above the cell so that the test strips were evenly lighted while a Logitech Pro 9000 USB camera was used to record images of the test strips as they were exposed to the flow of hydrogen peroxide vapor. A blue bandpass filter (Leitz Wetzlar BG 12) was placed between the test strips and the lens to restrict light throughput to 300–500 nm. This filter was selected experimentally during the work with nanoparticulate titania in a hydroxypropylcellulose matrix [33]. The work with the titanium(IV) test strips and liquid hydrogen peroxide (see discussed in Section 3.1, showed that the wider optical spectra of the light source combined with the color leakage between channels from a mosaic color filter array over the sensing element in the camera is lightly the reason for the improvement shown by the addition of the filter.

After the gas exits the PTFE cylinder, it is directed into a bubbler (labeled E) that contains 25 mL of the colorimetric hydrogen peroxide detection solution where a reaction occurs to produce a yellow color with an intensity that is proportional to the total amount of hydrogen peroxide passed into the bubbler. The gas from the first bubbler is passed through an optional second bubbler (not shown) as a backup in case hydrogen peroxide fails to be trapped in the first bubbler. Even when using 30% aqueous hydrogen peroxide as the source, there was no detectable color change in the second bubbler after 30 min. Thus, all hydrogen peroxide vapor was captured and complexed in the first bubbler. In accord with the OSHA colorimetric method for hydrogen peroxide [13], the concentration of the titanium peroxide complex was determined from the absorbance of the bubbler solution at 410 nm that was measured using a Cary 100 Bio UV-VIS spectrometer. A calibration curve was generated according to the specified OSHA method so that the concentration of captured hydrogen peroxide in the solution could be determined. The concentration of hydrogen peroxide vapor in the gas was calculated using this result, the volume of solution, and the gas flow rate and time.

The gas source bubbles through the hydrogen peroxide solution and can potentially be a source of water vapor, which could potentially dilute the solution in the final detection bubbles and affect the total volume of gas volume. An extended time (18 h) control experiment resulted in a maximum water mass loss of 0.5 g/h from the peroxide bubbler (labeled E). For an hour exposure, this mass of water corresponds to approximately 0.6 L of water vapor in 21 L of gas, given a maximum 3% error towards lower ppm. If all the water was deposited in the final bubbler, the liquid volume changed by only 2%. With a precision of less than 1 mL, no change in the water volume in the final bubbler is observed. Thus, the effect of water vapor on the calculated gas phase concentration of hydrogen peroxide is minimal.

### 2.4. Image Capture and Processing

The targeted gas phase concentration was obtained by varying the concentration of hydrogen peroxide in the bubbler solution. For precise values, some iteration is required due to variability in the stock hydrogen peroxide concentration with time. Once the gas flow was started, an image of the test strip was captured and saved every 30 s by a Python script using the OpenCV software package. Some care is required since buffering may result in an old image being returned by the framework. The work-around was to read the camera five times and take the last image. Experimentally, this resulted in consistent recording of the observed image. ImageJ, an open-source software package for processing and analyzing scientific images that was developed by the National Institutes of Health [34] was used to analyze the data collected from the test strips. It was found that the reflected blue light was too intense and saturated the blue channel. Therefore, the red channel was utilized since it has much lower sensitivity to blue light. To ensure the appropriate response, tests were performed using light at the desired analytical wavelengths generated using a broadband light source and a monochromator.

## 3. Results and Discussion

### 3.1. Response to Hydrogen Peroxide Solutions

The XploSafe PS test strips are designed to quantify the concentration of peroxides concentration when dipped into solutions containing them. The color of the test strip changes from white to yellow with the intensity of the final color increasing with solution concentrations. Thus, before the gas phase experiments were attempted, the solution response was first measured. For these experiments, each test strip was dipped into a set of standard hydrogen peroxide solutions (500, 350, 250, 100, 50 and 0 ppm by mass, accurate to less than 1%) for 1 min. After this, the excess solution was shaken off and the test strip image was taken within 10 min. It was found that the color was stable for at least 24 h in the laboratory environment. Although this process appears to be simple, there were multiple experimental issues that deserve commenting on. Some surprising issues were encountered in the procedure for the measurement of the reflected light and its division into three RGB (red, green, and blue) channels. To address these issues, a white reflectance standard was utilized to ensure that the images correctly represented the color of the object. First, it was determined that the light source was critical. It was found that LED bulbs and many of the fluorescent lights had significant issues with spectrum coverage. Indeed, some lightboxes for photographs are tinted toward yellow to mimic indoor lighting. Therefore, for both the solution and the gas phase experiments, a 20 W UV-filtered tungsten incandescent light was utilized. Secondly, the charge-coupled device (CCD) light detector was important. Most CCDs use a mosaic color filter array over the sensing element. It was determined that lower-cost cell phone cameras performed with significant color leakage between the RBG channels. Finally, it was found that post processing of the image by the camera or download software was unexpectedly problematic and must be avoided.

**Figure 4:**
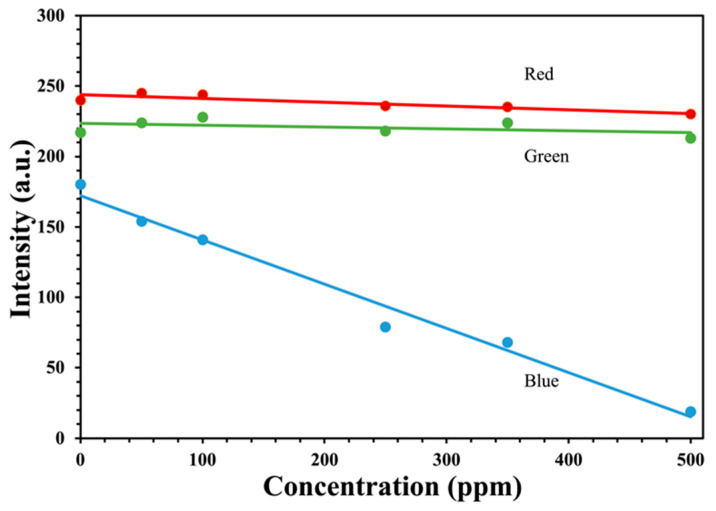
The red, green, and blue decomposition and intensities from reflected images of XploSafe PS test-strips after exposure to various concentrations of solution phase hydrogen peroxide. The color of the lines and markers represents each individual color channel.

Images were captured of each test strip using a 256-bit per channel single-lens reflex camera. The images were downloaded and processed in Corel PHOTO-PAINT. Each channel was separated and averaged over the pad of the test strip. Figure 4 shows the red, green, and blue (RGB) intensities at different concentrations of solution-phase hydrogen peroxide. The intensity at 0 ppm represents a test strip dipped into RO water. The color at 0 ppm is not white (the same intensity for each channel) even though the strip appears white to the human eye. This offset is either from the light source or the CCD detector of the camera and can be corrected using a reflection standard. As expected for the titanium(IV) peroxide complex, the absorption of light is in the blue region, resulting in a yellow color. Additionally, the resulting color changes matched the color scale provided by Xplosafe. Ultimately, this setup for optical measurement of the test strip response can be used to provide a significantly better quantification of the solution hydrogen peroxide concentration than that determined by visually comparing the test strip to a color chart.

### 3.2. Response to Hydrogen Peroxide Vapor

Upon exposure to gaseous hydrogen peroxide, the XploSafe PS test strips changed from colorless to yellow. As the test-strips react with the hydrogen peroxide vapor, the absorption of light occurs and less light is reflected. The detection of the reflected light is proportional to the concentration of titanium(IV) peroxide complex. Figure 3A,B show images before and after exposure to 28 ppm of hydrogen peroxide gas by volume for one hour, respectively. A blank test-strip did not have an observed color change during exposure to peroxide vapors. For these images, a Leiz Wetzlar BG 12 blue bandpass filter was placed before the camera. This minimized color leakage from the RGB filter on the CCD detector and mediated issues from the type of light source as the full color spectrum is no longer being detected. In addition, the outline of the unexposed strip is hard to see due to the bandpass filter. When the intensities were examined in detail, it was found that the images initially saturated the blue channel.

**Figure 5:**
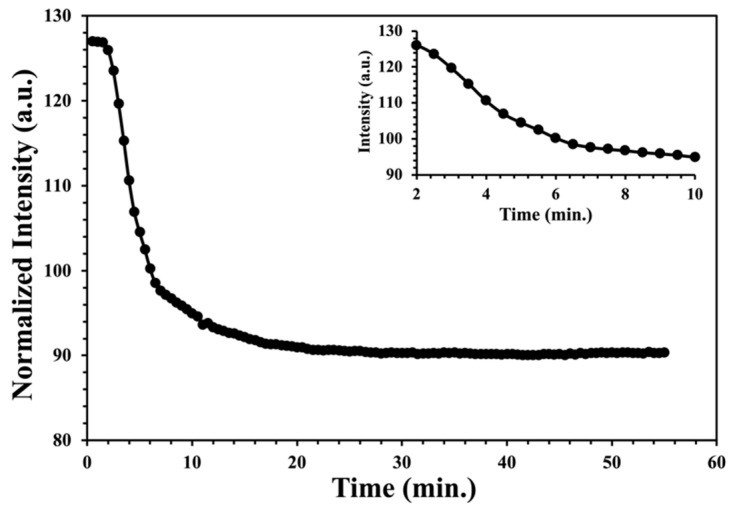
The raw reflected intensity with time from an XploSafe PS test strip during exposure to a hydrogen peroxide gas at a concentration of 28 ppm. The insert shows a blowup of rapid decrease in intensity between 2 and 10 min.

Test strips were exposed to hydrogen peroxide vapor for one hour at concentrations of 2.2, 11.6, 18.6, and 28.4 ppm by volume. For each exposure, images were taken every 30 s and saved to disk, as described in the experimental section. ImageJ was used to extract the intensity as a function of time from sequence of saved images. A typical decrease in raw reflected intensity with time is shown in Figure 5, with the inset showing a blow-up of the region of rapid decrease. The initial induction period (less than 2 min) is associated with the time required to reestablish the gas flow after introduction of the test strips. For each experimental data set, the hydrogen peroxide solution in the bubbler (Figure 2 Bubbler E) was adjusted to provide the required ppm in the gas phase. The gas phase ppm by volume was measured at the end of each run using the color change of the solution in the exit bubbler (Figure 2 Bubbler E) as decided in the experimental section.

**Figure 6:**
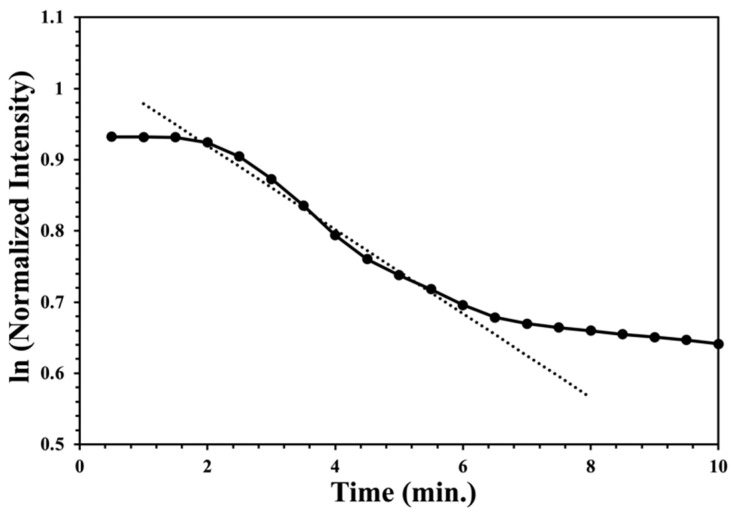
The natural logarithm of normalized reflected intensity with time from an XploSafe PS test-strip during the peroxide exposure to a hydrogen peroxide gas at a concentration of 28 ppm. The line is the linear regression used to determine the rate constant.

The intensity appears to exponentially decline with time during peroxide exposure. This observation was confirmed by plotting the natural logarithm of the normalized intensity versus exposure time (Figure 6), yielding a linear plot that is characteristic of an exponential time dependence. A linear regression can be performed for the data between 80% and 20% of the maximum intensity. All fits had an R^2^ greater than 0.90. The resulting negative slope is thus the phenomenological first-order rate constant. At later time points, the decrease in reflected intensity slows and then stops when all the accessible titanium(IV) is consumed. The linear fit implies that the color development is limited by the rate of arrival of hydrogen peroxide gas and the reaction between the hydrogen peroxide gas and the titanium(IV) species is fast on the time scale of this experiment. This result is consistent with the solution test method that relies on the fast, quantitative reaction between dissolved hydrogen peroxide and excess titanium(IV) oxysulfate in acidic solutions. Additionally, the gas phase peroxide concentration is held constant by the gas flow. The rates in units of seconds at the given ppm are summarized in Table 1. Error bars are computed from linear regression.

**Figure 7:**
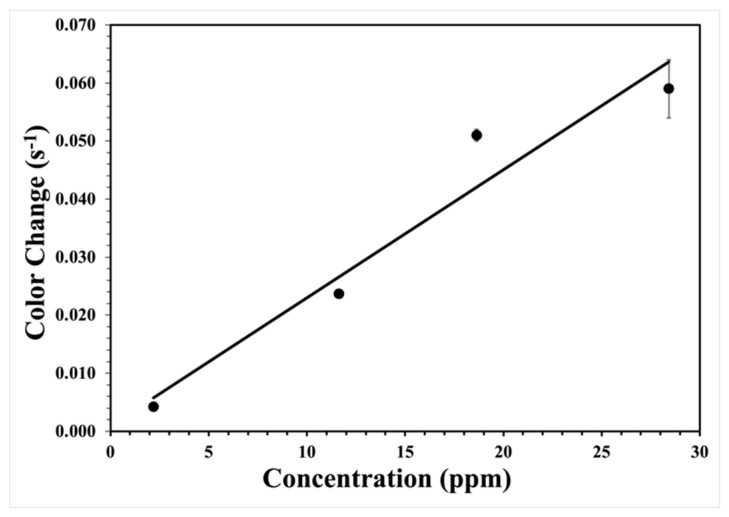
The phenomenological rate versus hydrogen peroxide concentration in ppm.

The response of the test strips with peroxide concentration can be quantified by plotting the rate constant versus the concentration. Each phenomenological first-order rate constant is the rate of color change with time at a given ppm. This rate is equal to the fixed concentration amount of titanium(IV) species times the arrival rate of hydrogen peroxide, which is proportional to its ppm. Thus, the measurement is the rate of color change per given ppm with time. Plotting the result verses ppm provides the color change per ppm per second. Figure 7 shows the resulting line. Independent of fitting through zero or not, the slope is 2.2 ± 0.3 × 10^−3^ color change per ppm per second. Assuming a conservative minimum detectable 5% color change from the initial intensity (see Figure 3A for reference), a detection limit of less than 1 ppm is possible for a one-minute exposure. For longer integration times, increased sensitivity is possible. For one-hour exposures, a detection limit of 0.01 ppm is computed. This result is similar but slightly better than the detection limit for titanium(IV) hydroxypropyl cellulose films previously measured using similar methods [33].

## 4. Conclusions

This paper shows that the combination of Xplosafe PS test strip with a low-cost camera can quantify hydrogen peroxide with high sensitivity in the liquid and gas phases. In both cases, the exposure of titanium(IV) oxysulfate to hydrogen peroxide leads to the development of a characteristic intense yellow color. For liquids, the test strips were tested with solution concentrations between 50 and 500 ppm by mass. The liquid reaction was completed in less than one minute, and the final color change was quantified using a digital single-lens reflex camera. Color leakage due to the RGB filter on the CCD detector and issues with the light source were issues and required careful selection of the equipment and the method of data analysis. If done properly, RGB color separation and analysis can provide more accurate results than simple visual comparison to a color chart. The results of an unmodified commercial camera to quantify a test strip with proper lighting show clear potential as a quantitative method.

For hydrogen peroxide gas sensing, an inexpensive web camera and a tungsten lamp were used to measure the reflected light intensity as a function of exposure from a test strip. The use of a blue filter provided a simple way to increase the sensitivity. First-order behavior in the color change with exposure time was observed during the exposure to peroxide vapor over a range of peroxide concentration from 2 to 30 ppm by volume, implying that the reaction between the titanium(IV) species in the test strips with the arriving hydrogen peroxide molecules is rapid on the time scale of these experiments. The change in intensity is linearly proportional to the concentration of the gas phase hydrogen peroxide. The resulting color change is 2.2 ± 0.3 × 10^−3^ color change per ppm per second. The gas-phase detection limit was estimated to be less than 1 ppm after a one-minute measurement and 0.01 ppm after a one-hour integration. The stability of the titanium(IV) complex allows passive time-integrated exposure measurements for increased sensitivity. Thus, titanium(IV) oxysulfate based test strips have high potential for low-cost workplace hydrogen peroxide monitoring.

## Figures and Tables

**Table 1:** The phenomenological first-order rate constants and error bars determined at different hydrogen peroxide ppm.

Hydrogen Peroxide (ppm by Volume)	Rate Constant (s^−1^)	R^2^
28.4	0.059 ± 0.005	0.98
18.6	0.051 ± 0.001	0.99
11.6	0.0237 ± 0.0005	0.99
2.2	0.0042 ± 0.0003	0.98

## Data Availability

The data presented in this study are available on request from the corresponding author.
